# Microsomal Prostaglandin E Synthase-1 Deficiency Exacerbates Pulmonary Fibrosis Induced by Bleomycin in Mice

**DOI:** 10.3390/molecules19044967

**Published:** 2014-04-21

**Authors:** Bo Wei, Linhong Cai, Dan Sun, Yanhua Wang, Cairui Wang, Xiaoyu Chai, Feng Xie, Ming Su, Fangrui Ding, Jie Liu, Jichun Yang, Youfei Guan, Xinmin Liu

**Affiliations:** 1 Department of Geriatrics, Peking University First Hospital, Peking University, Beijing 100034, China; E-Mails: weibo-0@126.com (B.W.); shelley007810626@hotmail.com (D.S.); wangyanhua3826@163.com (Y.W.); queen-rui@163.com (C.W.); klmytchristy@163.com (X.C.); 2 No.3 Outpatient Department, No.58 Anli Road, Chaoyang District, Beijing 100021, China; E-Mail: philk_515@163.com; 3 Department of Urology, Peking University First Hospital & the Institute of Urology, Peking University, Beijing 100034, China; E-Mails: xiefengjoy@126.com (F.X.); soloriver@126.com (J.L.); 4 National Urological Cancer Center, Beijing 100034, China; 5 Sino-German Laboratory for Molecular Medicine, State Key Laboratory of Cardiovascular Disease, FuWai Hospital & Cardiovascular Institute, Chinese Academy of Medical Sciences, Peking Union Medical College, Beijing 100037, China; E-Mail: suming28@163.com; 6 Department of Pediatrics, Peking University First Hospital, Peking University, Beijing 100034, China; E-Mail: youngbear@126.com; 7 Department of Physiology and Pathophysiology, Peking University School of Basic Medical Sciences, 38 Xueyuan Road, Beijing 100191, China; E-Mail: yangj@hsc.pku.edu.cn; 8 MOE Key Lab of Molecular Cardiovascular Science, Peking University, 38 Xueyuan Road, Beijing 100191, China; 9 Department of Physiology and Pathophysiology, Peking University Health Science Center, Beijing 100191, China; E-Mail: youfeiguan@szu.edu.cn

**Keywords:** microsomal prostaglandin E synthase-1, idiopathic pulmonary fibrosis, prostaglandin E2, E prostanoid receptor 2, focal adhesion kinase

## Abstract

Microsomal prostaglandin E2 synthase-1 (mPGES-1), an inducible enzyme that converts prostaglandin H2 (PGH_2_) to prostaglandin E2 (PGE_2_), plays an important role in a variety of diseases. So far, the role of mPGES-1 in idiopathic pulmonary fibrosis (IPF) remained unknown. The current study aimed to investigate the role of mPGES-1 in pulmonary fibrosis induced by bleomycin in mice. We found that mPGES-1 deficient (mPGES-1^−/−^) mice exhibited more severe fibrotic lesions with a decrease in PGE_2_ content in lungs after bleomycin treatment when compared with wild type (mPGES-1^+/+^) mice. The mPGES-1 expression levels and PGE_2_ content were also decreased in bleomycin-treated mPGES-1^+/+^ mice compared to saline-treated mPGES-1^+/+^ mice. Moreover, in both mPGES-1^−/−^ and mPGES-1^+/+^ mice, bleomycin treatment reduced the expression levels of E prostanoid receptor 2 (EP2) and EP4 receptor in lungs, whereas had little effect on EP1 and EP3. In cultured human lung fibroblast cells (MRC-5), siRNA-mediated knockdown of mPGES-1 augmented transforming growth factor-β1 (TGF-β1)-induced α-smooth muscle actin (α-SMA) protein expression, and the increase was reversed by treatment of PGE_2_, selective EP2 agonist and focal adhesion kinase (FAK) inhibitor. In conclusion, these findings revealed mPGES-1 exerts an essential effect against pulmonary fibrogenesis via EP2-mediated signaling transduction, and activation of mPGES-1-PGE_2_-EP2-FAK signaling pathway may represent a new therapeutic strategy for treatment of IPF patients.

## 1. Introduction

Idiopathic pulmonary fibrosis (IPF) is a chronic, progressive disease primarily affecting elderly adults and characterized by the proliferation of lung fibroblasts and excessive collagen deposition [1]. Although the mechanism of IPF remains yet largely unknown, several factors such as inflammation, coagulation, oxidative stress, and epithelial mesenchymal transition (EMT) have been proposed to play important roles in the development and progression of IPF [2]. Typical histopathological changes of pulmonary fibrosis include alveolar epithelial cell injury, and differentiation of fibroblasts to myofibroblasts. An increase in α-smooth muscle actin (α-SMA) expression is the central event in the differentiation of fibroblasts to myofibroblasts [3]. Hydroxyproline is one of the main components in the collagen protein, with the content levels correlating with collagen deposition and the severity of pulmonary fibrosis [4]. While no large-scale etiological studies relating to factors such as geography, ethnicity or racial factors have been performed, it is clear that IPF cases are on the rise [5]. Thus, further research on its pathogenesis and the development of novel therapeutic strategies are required to meet this medical need.

Prostaglandins (PGs) are important internal inflammation mediators, and their abnormal production has been shown to tightly be associated with a variety of diseases. Prostaglandin E2 (PGE_2_), a member of PG family, is synthesized when cyclooxygenase (COX) induces PGG_2_ to PGH_2_ [6], followed by the prostaglandin E (PGE) synthase mediated conversion of PGH_2_ to PGE_2_ [7]. Three types of PGE synthases controlling PGE_2_ production in cells have been identified before. Two are membrane-associated, which are designated as mPGES-1 and mPGES-2, respectively. The third is cytosolic, which is designated as cPGES [7,8]. mPGES-2 is constitutively expressed in many tissues [9], whereas mPGES-1 expression is induced in response to inflammation [10,11]. So far, mPGES-1 has been demonstrated to be directly associated with many diseases such as pain, fever, tumorigenesis, atherosclerosis, reproduction and skin fibrogenesis [12,13,14,15,16,17], yet there is no current research pertaining to the function of mPGES-1 in IPF. In addition, IPF is a typical disease related with inflammation, and previous research has proved PGE_2_ production was decreased in lung fibroblasts isolated from IPF patients [18], so we hypothesize that mPGES-1, one of PGE synthases, may play an essential role in IPF development and progression.

Bleomycin, an anti-tumor antibiotic isolated initially from the fungus *Streptomyces verticillus* [19], is extensively used in induction of pulmonary fibrosis in animal models [20]. Moreover, fibroblasts stimulated by transforming growth factor-β1 (TGF-β1) differentiate into myofibroblasts, from which extensive extracellular matrix is accumulated to form lung fibrosis [21]. In this study, we used these methods to investigate the function of mPGES-1 in pulmonary fibrosis in order to further clarify the underlying mechanisms and to search for a new target for the treatment of IPF.

## 2. Results and Discussion

### 2.1. Results

2.1.1. mPGES-1^−/−^ Mice Exhibited More Severe Lung Fibrosis after Bleomycin Treatment

**Figure 1:**
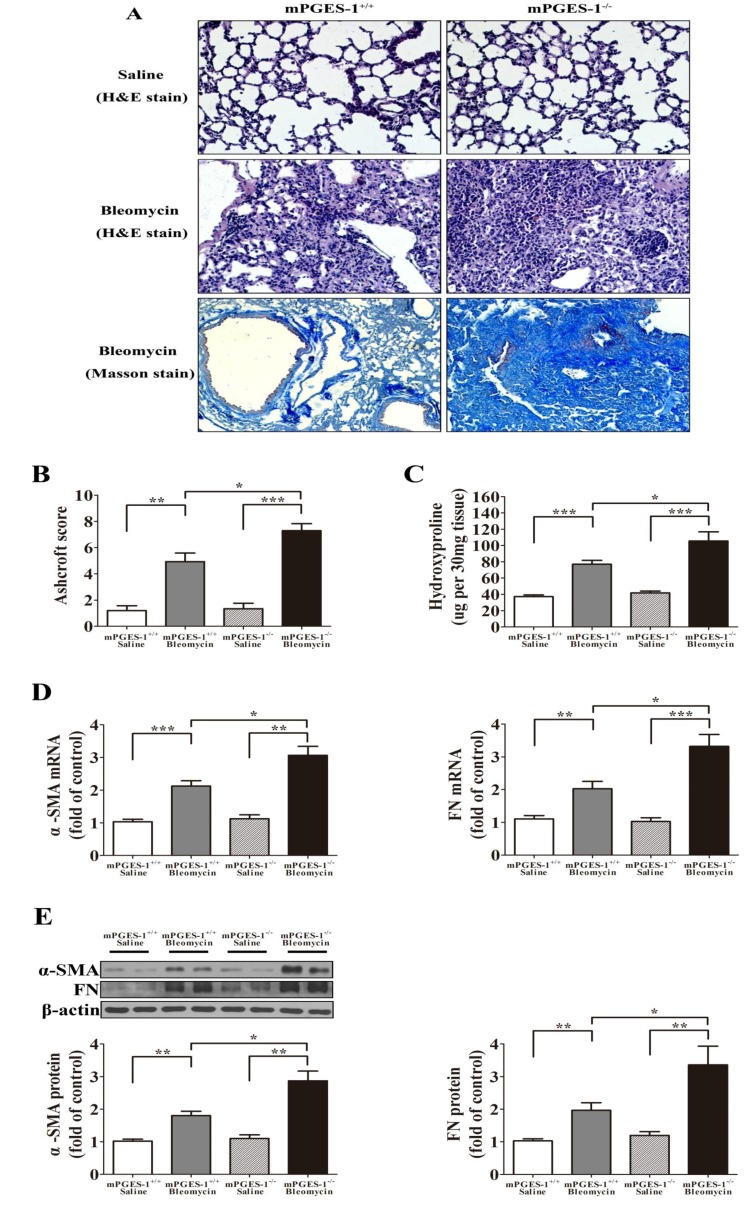
mPGES-1 deficient mice exhibited more severe lung fibrotic injury following bleomycin treatment. (**A**) Representative histological changes from each group showing increased lung lesions and inflammation in the mPGES-1^−/−^ mice receiving bleomycin when compared with wild type group. Lungs were stained with hematoxylin and eosin (H&E) staining (magnification: ×200) or Masson staining (magnification: ×100); (**B**) Semi-quantitative assessment with the Aschroft score method was made on day 28 post administration, with a significantly higher score observed in the mPGES-1^−/−^ mice with bleomycin treatment when compared with mPGES-1^+/+^ mice with bleomycin treatment; (**C**) The hydroxyproline content in the lung was significantly higher in mPGES-1^−/−^ mice with bleomycin treatment when compared with the mPGES-1^+/+^ mice with bleomycin treatment. (**D**,**E**) Assessment of α-SMA and FN mRNA and protein expression from each group on day 28 after administration. (**D**) Statistical chart of mRNA expression. (**E**) Representative western blot images and scanning densitometry of protein expression. Results are expressed as means ± SEM (*n* = 4–6 mice per group) (* *p* < 0.05, ** *p* < 0.01, *** *p* < 0.001).

Histopathological evaluation of paraffin-embedded lung sections was examined to establish lung fibrosis. While no morphological changes were observed in mPGES-1^+/+^ and mPGES-1^−/−^ treated with saline, significant fibrotic changes were noted in bleomycin-treated lung samples. The mPGES-1^+/+^ mice with bleomycin displayed moderate fibrotic lesions, inflammatory cell infiltration, thickening of the interstitium, and contained moderate collagen deposition. Furthermore, the mPGES-1^−/−^ mice with bleomycin exhibited more severe fibrosis characterized by increased inflammatory cell infiltration, a complete loss of alveolar architecture and massive collagen deposition resulting in enhanced fibrosis (Figure 1A).

The grades of fibrosis were determined utilizing the Ashcroft scoring method. The fibrosis scores for mPGES-1^+/+^ mice with saline is 1.20 ± 0.36, mPGES-1^+/+^ mice with bleomycin is 4.93 ± 0.66, mPGES-1^−/−^ mice with saline is 1.34 ± 0.42 and mPGES-1^−/−^ mice with bleomycin is 7.30 ± 0.54. A significant increase in the two bleomycin**-**treated groups relative to the saline**-**treated groups was noted (*p* *<* 0.01), with the scores for the mPGES-1^−/−^ mice with bleomycin group significantly elevated when compared with the mPGES-1^+/+^ mice with bleomycin group (*p* *<* 0.05) (Figure 1B).

Hydroxyproline content was quantified to reflect collagen deposition in the lungs as a means to assess the extent of lung fibrosis for each experimental group. Hydroxyproline content was assessed as μg per 30 mg tissue sample and the values in four groups of mice were as following: mPGES-1^+/+^ mice with saline (37.14 ± 2.08), mPGES-1^+/+^ mice with bleomycin (76.93 ± 4.81), mPGES-1^−/−^ mice with saline (41.81 ± 2.30) and mPGES-1^−/−^ mice with bleomycin (105.4 ± 11.08). A significant increase in hydroxyproline content was noted in bleomycin treated samples when compared with groups treated with saline (*p* *<* 0.001). Importantly, the hydroxyproline content of the mPGES-1^−/−^ mice receiving bleomycin was significantly increased when compared with the mPGES-1^+/+^ mice receiving bleomycin (*p* *<* 0.05) (Figure 1C).

The mRNA and protein expression levels of α-SMA and fibronection (FN) in bleomycin treated samples were increased when compared to saline treated samples (*p* *<* 0.01). Notably, α-SMA and FN expression levels were significantly higher in mPGES-1^−/−^ mice receiving bleomycin when compared with mPGES-1^+/+^ mice with bleomycin (*p* *<* 0.05) (Figure 1D–E).

#### 2.1.2. Bleomycin Treatment Reduced mPGES-1 Expression Levels in mPGES-1^+/+^ Mice, Inhibited PGE_2_ Production whereas Increased LTC_4_ Content in the Lungs from both mPGES-1^+/+^ and mPGES-1^−/−^ Mice

**Figure 2:**
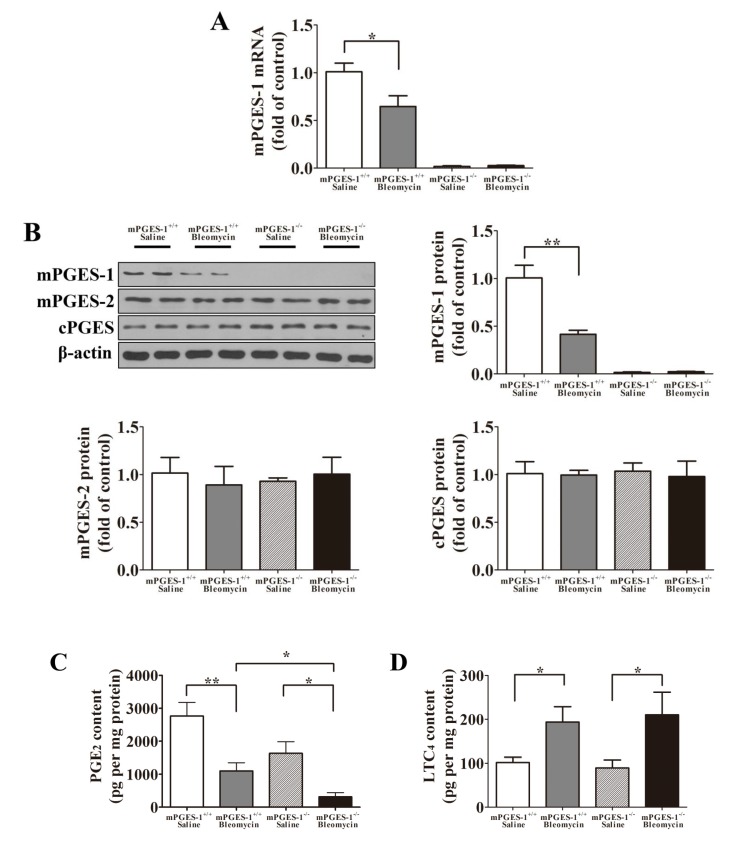
The mRNA and protein levels of mPGES-1, mPGES-2 and cPGES in addition to PGE_2_ content in the lungs of mice on day 28 after instillation. (**A**) Statistical chart of mPGES-1 mRNA expression; (**B**) Representative western blot images and scanning densitometry of mPGES-1, mPGES-2 and cPGES protein expression; (**C**) The PGE_2_ content in the lungs of mPGES-1^+/+^ mice and mPGES-1^−/−^ mice receiving bleomycin or saline; (**D**) The LTC_4_ content in the lungs of mPGES-1^+/+^ mice and mPGES-1^−/−^ mice receiving bleomycin or saline. Results are expressed as means ± SEM (*n* = 4–6 mice per group) (* *p* < 0.05, ** *p* < 0.01).

Bleomycin treatment reduced the mRNA and protein levels of mPGES-1 in the lungs of the mPGES-1^+/+^ mice when compared with saline-treated mice (*p* *<* 0.05). As expected, both the mRNA and protein of mPGES-1 were not present in the lungs of mPGES-1^−/−^ mice (Figure 2A,B). The protein expression levels of mPGES-2 and cPGES were not significantly different in mPGES-1^−/−^ and mPGES-1^+/+^ mice treated with saline or bleomycin (Figure 2B). The PGE_2_ content in four groups of mice was as following: the mPGES-1^+/+^ mice with saline (2763 ± 415.2 pg per mg protein), mPGES-1^+/+^ mice with bleomycin (1101 ± 249.6 pg per mg protein), mPGES-1^−/−^ mice with saline (1631 ± 353.9 pg per mg protein) and mPGES-1^−/−^ mice with bleomycin (312.8 ± 128.5 pg per mg protein). Although bleomycin treatment reduced PGE_2_ content in the lungs from both mPGES-1^−/−^ and mPGES-1^+/+^ mice, it resulted in a more pronounced reduction in the mPGES-1^−/−^ mice (*p* *<* 0.05) (Figure 2C). The LTC_4_ content in four groups of mice was as following: the mPGES-1^+/+^ mice with saline (101.8 ± 11.94 pg per mg protein), mPGES-1^+/+^ mice with bleomycin (193.5 ± 35.15 pg per mg protein), mPGES-1^−/^^−^ mice with saline (89.30 ± 18.04 pg per mg protein) and mPGES-1^−/^^−^ mice with bleomycin (210.2 ± 51.52 pg per mg protein) (*p* *<* 0.05) (Figure 2D). This data indicates that mPGES-1 deficiency does not exacerbate bleomycin-induced lung fibrosis in mice via 5-lipoxygenase (5-LO) metabolic pathway.

#### 2.1.3. Bleomycin Treatment Reduced the Expression of EP2 and EP4 in mPGES-1^−/−^ and mPGES-1^+/+^ Mice Lungs

**Figure 3:**
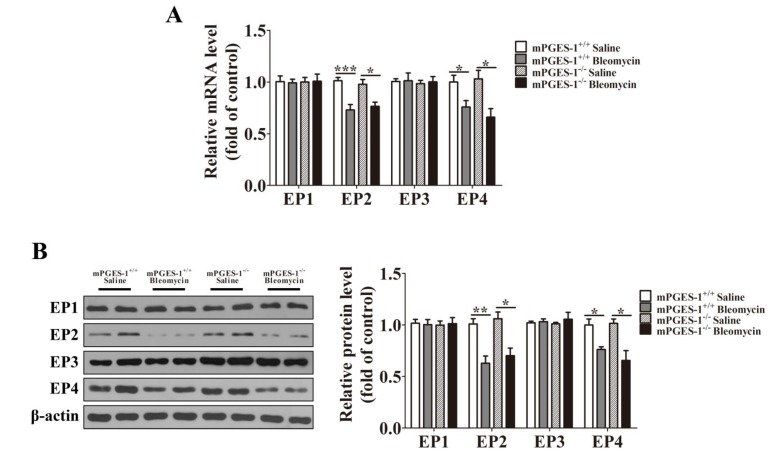
The expression of PGE_2_ receptor subtypes in the lungs of mPGES-1^+/+^ mice and mPGES-1^−/−^ mice receiving bleomycin or saline. (**A**) Statistical chart of mRNA expression; (**B**) Representative western blot images and scanning densitometry of protein expression. Results are expressed as means ± SEM (*n* = 4–6 mice per group) (* *p* < 0.05, ** *p* < 0.01, *** *p* < 0.001).

Both the mRNA and protein expression levels of EP2 and EP4 were significantly decreased in the lungs of mPGES-1^−/−^ and mPGES-1^+/+^ mice treated with bleomycin when compared with saline (*p* *<* 0.05). However, bleomycin treatment failed to affect the expression of EP1 and EP3. In the absence or presence of bleomycin treatment, the expression levels of EP1, EP2, EP3 and EP4 were not significantly different between mPGES-1^−/−^ and mPGES-1^+/+^ mice (Figure 3). These findings suggested that a reduction of EP2 and EP4 expression might be involved in pulmonary fibrosis induced by bleomycin in mPGES-1^−/−^ and mPGES-1^+/+^ mice.

#### 2.1.4. Aggravation of Bleomycin-Induced Lung Fibrosis in mPGES-1^−/−^ Mice Was not Dependent on Smad2/3 Pathway

**Figure 4:**
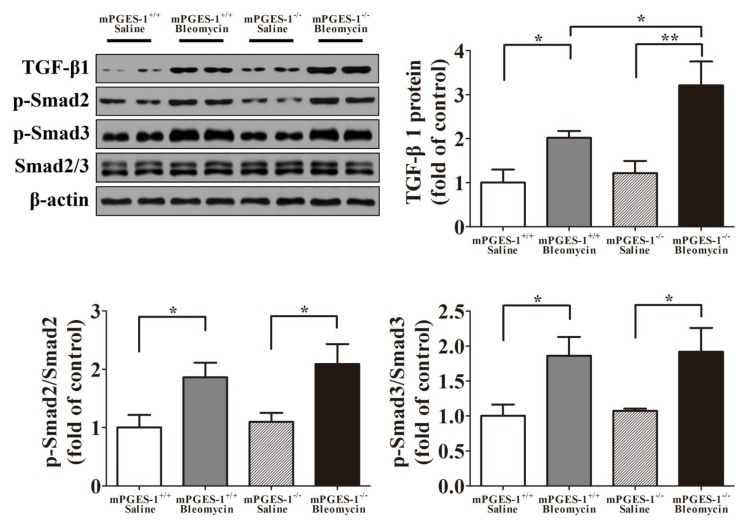
The representative western blot images and scanning densitometry of protein expression of TGF-β1 and p-Smad2/3 in the lungs of mPGES-1^+/+^ and mPGES-1^−/−^ mice after administration. Results are expressed as means ± SEM (*n* = 4–6 mice per group) (* *p* < 0.05, ** *p* < 0.01).

Western blotting analysis showed that the protein expression levels of TGF-β1 and p-Smad2/3 were significantly increased in the lungs of mPGES-1^−/−^ and mPGES-1^+/+^ mice treated with bleomycin when compared with saline (*p* *<* 0.05). Furthermore, TGF-β1 expression levels were significantly higher in mPGES-1^−/−^ mice than in mPGES-1^+/+^ mice with bleomycin. However, the protein expression levels of p-Smad2/3 did not differ between mPGES-1^−/−^and mPGES-1^+/+^ mice after bleomycin treatment (Figure 4). These findings suggested that exacerbation of bleomycin-induced lung fibrosis in mPGES-1^−/−^ mice was independent on Smad2/3 pathway.

#### 2.1.5. TGF-β1 Treatment Reduced the Expression of mPGES-1, EP2 and EP4 in MRC-5 Cells

**Figure 5:**
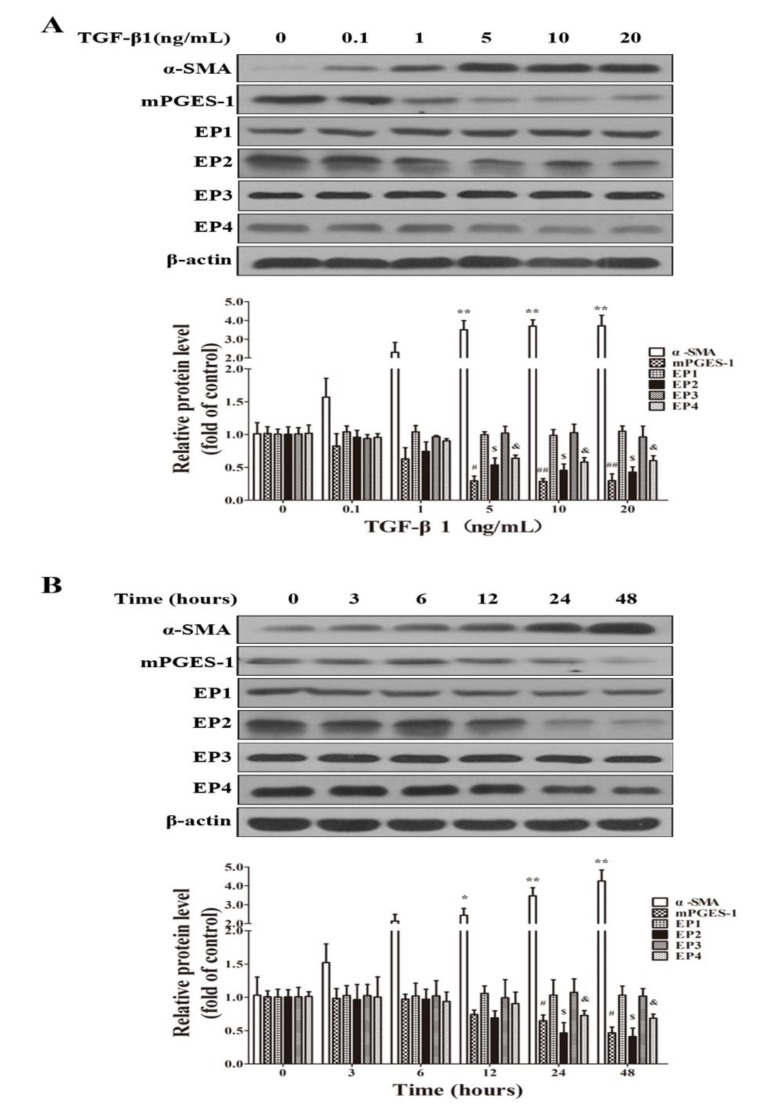
TGF-β1 treatment reduced mPGES-1, EP2 and EP4 protein expression in MRC-5 cell lines. When the cells were grown to 80% confluence, they were starved for 24 h and treated with various concentrations of TGF-β1 (0, 0.1, 1, 5, 10 and 20 ng/mL) for 24 h or treated with 5 ng/mL TGF-β1 for various time length (0, 3, 6, 12, 24 and 48 h). TGF-β1 dose-dependently increased the protein levels of α-SMA, and reduced that of mPGES-1, EP2 and EP4, and the representative western blot images and scanning densitometry of protein expression were shown in (**A**); (**B**) Time course of TGF-β1 on the expression levels of α-SMA, mPGES-1, EP1, EP2, EP3 and EP4 in MRC-5 cells. Results are expressed as means ± SEM (*n* = 3) (* *p* < 0.05, ** *p* < 0.01 *versus* TGF-β1 absence or TGF-β1 treated at 0 time point group in α-SMA expression level; ^#^ *p* < 0.05, ^##^ *p* < 0.01 *versus* TGF-β1 absence or TGF-β1 treated at 0 time point group in mPGES-1 expression level; ^$^ *p* < 0.05 *versus* TGF-β1 absence or TGF-β1 treated at 0 time point group in EP2 expression level; ^&^ *p* < 0.05 *versus* TGF-β1 absence or TGF-β1 treated at 0 time point group in EP4 expression level).

TGF-β1 was used to establish a fibrosis model in MRC-5 cell lines. In MRC-5 cells, TGF-β1 treatment significantly induced α-SMA expression, which is one of the main markers for fibrosis in lungs and other tissues or cells. Importantly, in MRC-5 cells treated with TGF-β1, with an increase in α-SMA expression, the expression levels mPGES-1, EP2 and EP4 were reduced. In contrast, the expression levels of EP1 and EP3 were not significantly affected by TGF-β1 in MRC-5 cells (Figure 5).

**Figure 6:**
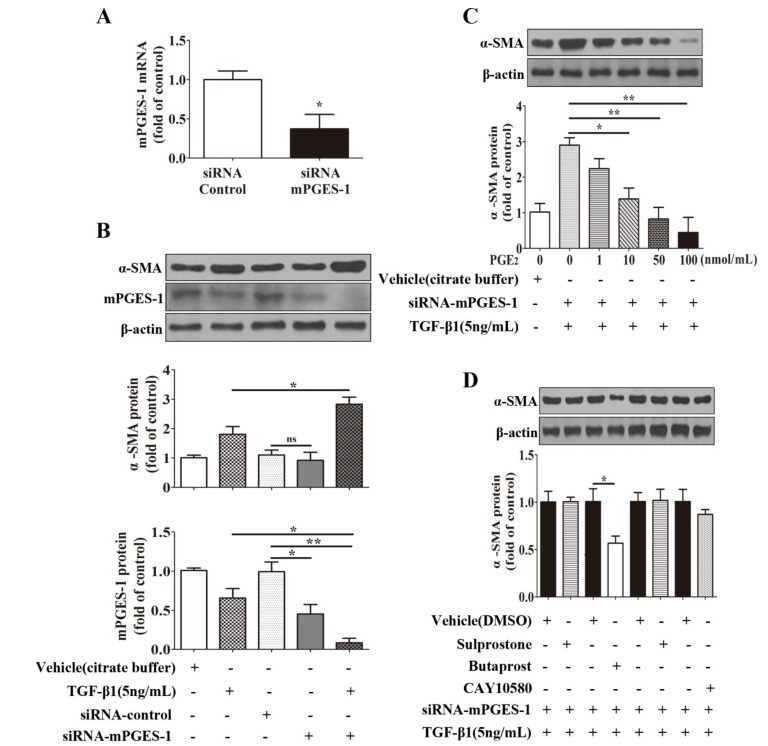
mPGES-1-derived PGE_2_ exerts a beneficial effect against α-SMA expression induced by TGF-β1 in MRC-5 cells via EP2 receptor-mediated signaling transduction. After starvation and siRNA transfection, the cells were co-incubated with TGF-β1 and various concentrations of PGE_2_ (0, 1, 10, 50, 100 nmol/mL) for 24 hours or with TGF-β1 and various EP agonists (sulprostone for EP1 and EP3, butaprost for EP2, and CAY10580 for EP4, 100 nmol/mL) for 24 h. (**A**) siRNA treatment on mPGES-1 mRNA level in MRC-5 cells; (**B**) Knockdown of mPGES-1 augmented TGF-β1’s stimulatory effect on α-SMA protein expression;(**C**) PGE_2_ dose-dependently inhibited α-SMA protein expression induced by TGF-β1 in MRC-5 cells with mPGES-1 siRNA transfection; (**D**) Butaprost, EP2 agonist, decreased α-SMA protein expression induced by TGF-β1 in MRC-5 cells with mPGES-1 siRNA transfection. Results are expressed as means ± SEM (*n* = 3) (* *p* < 0.05, ** *p* < 0.01, ns, not significant).

#### 2.1.6. mPGES-1-Derived PGE_2_ Exerts a Beneficial Effect against α-SMA Expression Induced by TGF-β1 in MRC-5 Cells via EP2 Receptor-Mediated Signaling Transduction

To further study the role of mPGES-1-drived PGE_2_ in fibrosis, its expression was knockdown by small interfering RNA (siRNA) against mPGES-1 mRNA. mPGES-1 siRNA treatment significantly reduced the mRNA and protein levels of mPGES-1 by about 50% in MRC-5 cells (Figure 6A,B). In the absence of TGF-β1 stimulation, knockdown of mPGES-1 had no significant effect on α-SMA expression. However, knockdown of mPGES-1 significantly augmented TGF-β1’s stimulatory effect on α-SMA expression in MRC-5 cells (Figure 6B). Importantly, PGE_2_ dose-dependently reduced TGF-β1-induced increase in α-SMA expression in MRC-5 cells with mPGES-1 knockdown (Figure 6C). These results suggested that a reduction in mPGES-1 derived PGE_2_ is involved in TGF-β1-induced α-SMA expression in MRC-5 cells.

To determine which subtype of PGE_2_ receptors mediate PGE_2_’s protective effect against TGF-β1-induced cellular fibrosis, the cells with mPGES-1 knockdown was treated with agonists of EP1, EP2, EP3, and EP4 in the presence TGF-β1 stimulation in MRC-5 cells. The results clearly indicated that in MRC-5 cells with mPGES-1 knockdown, only the agonist of EP2, butaprost, reduced TGF-β1-induced α-SMA expression. In contrast, sulprostone, the dual agonist for EP1 and EP3, and CAY10580, the agonist for EP4, failed to affect TGF-β1-induced α-SMA expression (Figure 6D). These findings revealed that mPGES-1-derived PGE_2_ exerts a beneficial effect against α-SMA expression via EP2 receptor signaling pathway.

#### 2.1.7. mPGES-1-Derived PGE_2_ Exerts a Beneficial Effect against α-SMA Expression Induced by TGF-β1 in MRC-5 Cells via Inhibition of FAK Autophosphorylation

**Figure 7:**
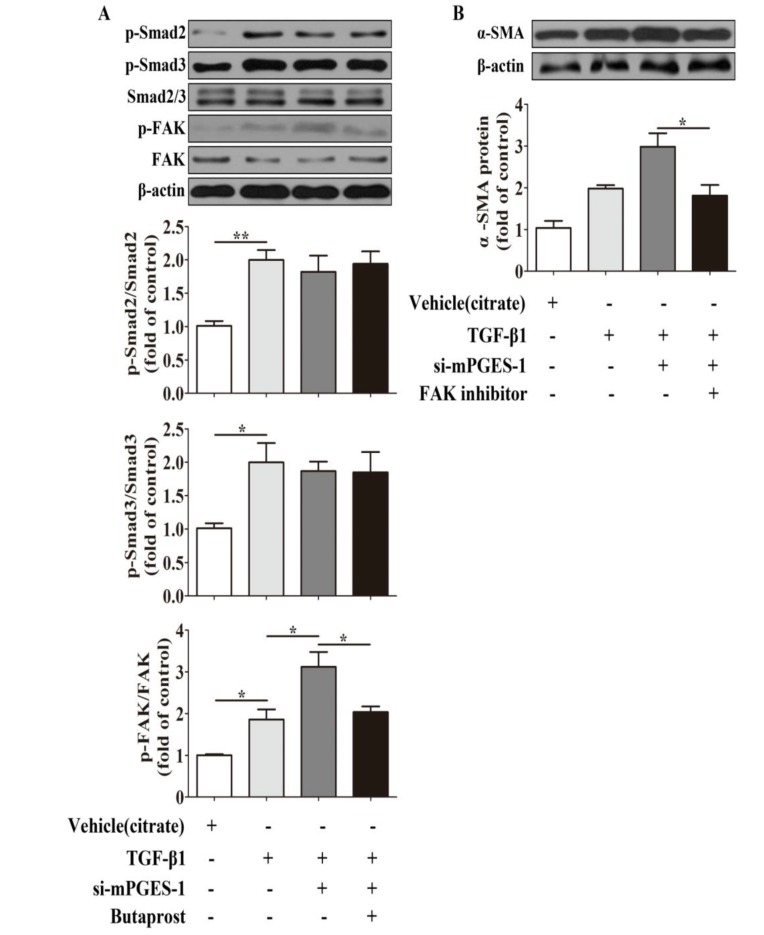
mPGES-1-derived PGE_2_ Exerts a Beneficial Effect against α-SMA Expression Induced by TGF-β1 in MRC-5 Cells via inhibition of FAK autophosphorylation. After starvation and siRNA transfection, the cells were co-incubated with TGF-β1 and EP2 agonist (butaprost, 100 nmol/mL) for 24 h or with TGF-β1 and FAK inhibitor (FAK inhibitor 14, 10 μmol/mL) for 24 h. (**A**) Butaprost, EP2 agonist, decreased p-FAK protein expression induced by TGF-β1 in MRC-5 cells with mPGES-1 siRNA transfection; (**B**) Inhibition of FAK autophosphorylation reduced α-SMA protein expression after TGF-β1 and mPGES-1 siRNA co-incubation in MRC-5 cells. Results are expressed as means ± SEM (*n* = 3) (* *p* < 0.05, ** *p* < 0.01, si-mPGES-1, siRNA-mPGES-1).

Since other studies have demonstrated that focal adhesion kinase (FAK) autophosphorylation is involved in the inhibition of PGE_2_ to myofibroblasts differentiation induced by TGF-β1 [22], we observed the effect of EP2 agonist on FAK autophosphorylation mediated by TGF-β1 and mPGES-1 siRNA co-incubation, and the effect of FAK inhibitor on TGF-β1-induced α-SMA expression to further explore the potential link of mPGES-1-PGE_2_-EP2 axis and fibrosis. We found that butaprost could weaken p-FAK, but not p-Smad2/3 expression treated by TGF-β1 and mPGES-1 siRNA (Figure 7A). Moreover, FAK inhibitor attenuated α-SMA expression induced by TGF-β1 and mPGES-1 siRNA (Figure 7B). These findings indicated that mPGES-1-PGE_2_-EP2 axis’s beneficial effect against TGF-β1-induced α-SMA expression via inhibition of FAK autophosphorylation.

### 2.2. Discussion

In the current study, we found that bleomycin-treatment resulted in more notable histopathological changes, higher hydroxyproline content and increased mRNA and protein expression of α-SMA in mPGES-1^−/−^ mice than in wild type mice. We further demonstrated beyond inhibition of PGE_2_ production from mPGES-1, bleomycin also repressed the expression of PGE_2_ receptors EP2 and EP4. These findings strongly suggested that inhibitoin of mPGES-1-PGE_2_-EP2/EP4 signaling axis plays an important role in bleomycin-induced IPF. Furthermore, we found that only the agonist of EP2 can inhibit TGF-β1-induced increase in α-SMA expression in MRC-5 cells with mPGES-1 knockdown. Clearly, these findings revealed that mPGES-1-derived PGE_2_ exerts a beneficial effect against pulmonary fibrosis via EP2 receptor-mediated signal transduction.

mPGES-1, a terminal synthase of PGE_2_, has been reported as a key mediator in a variety of diseases. To our knowledge, little is known about the role of mPGES-1 in pulmonary fibrogenesis up to now. McCann *et al* reported that mPGES-1 expression is increased in bleomycin-induced skin fibrogenesis. mPGES-1 deficient mice were resistant in bleomycin-induced skin fibrogenesis. These findings suggested that mPGES-1 is required in the development of skin fibrogenesis [17]. In the present study, we found that deficiency of mPGES-1 aggravated bleomycin-induced injury and pulmonary fibrosis. Currently, the role of inflammation mediators such as PGE_2_ in the pathogenesis of fibrosis is controversial, with this lack of clarity possibly attributed to mPGES-1 operating via tissue specific mechanisms.

In healthy lung tissue, microenvironment homeostasis is dependent on alveolar epithelial cell (AEC) and mesenchymal cell (MSC) crosstalk [23]. The ability of the AECs to generate PGE_2_ is critical for blocking fibroblast proliferation [24,25,26]. In consistent with our observations, other studies also revealed that lung fibroblasts from IPF patients had reduced PGE_2_ levels [18]. In our study, we found that bleomycin treatment reduced PGE_2_ content in the lungs of wild type mice. Notably, bleomycin treatment resulted in a marked reduction in lung PGE_2_ levels in mPGES-1^−/−^ mice. We also found that when compared to mPGES-1^+/+^ mice, mPGES-1^−/−^ mice only exhibited a modest reduction in cellular PGE_2_ levels in the lungs without bleomycin treatment. These findings may suggest that other two types of PGE_2_ synthase, mPGES-2 and cPGES, play important roles in controlling PGE_2_ production in the basic condition. In support, the expression levels of mPGES-2 and cPGES remained unchanged in the lungs of mPGES-1^+/+^ mice and mPGES-1^−/−^ mice receiving belomycin or saline (Figure 2B). Overall, these findings strongly suggested that a reduction in mPGES-1 expression, leading to the inhibition of PGE_2_ synthesis, plays a unique and important role in IPF induced by bleomycin.

PGE_2_ activates its downstream pathways through 4 different subtypes of receptors, which are designated as EP1 (E prostanoid receptors 1), EP2, EP3 and EP4, respectively, in various tissues [27]. It had been previously reported that PGE_2_ exerts anti-fibrotic actions through EP2 and EP4 pathways *in vitro.* The activation of these pathways increased cellular cyclic adenosine monophosphate (cAMP) level, the activation of protein kinase A (PKA), or exchange protein, leading to the repression of matrix proteins expression and blockade of the proliferation of fibroblasts [28,29]. In fibrotic lungs, the expression levels of EP receptor subtypes are altered. Similar to our findings, it had also been previously reported that EP2 expression was decreased in bleomycin-induced fibrotic mice, leading to increased fibroblast proliferation and decreased matrix proteins degradation [28]. Huang SK *et al.* confirmed that there was some variable PGE_2_ resistance in fibroblast from usual interstitial pneumonia patients and the reason for that was partly due to decreased expression of EP2 and PKA [30]. In our experiment, we found that EP2 and EP4 expression levels was reduced in lungs from mice after bleomycin treatment, and human lung fibroblasts induced by TGF-β1. Furthermore, we observed the fibroblast to myofibroblast differentiation was inhibited by butaprost, EP2 agonist, but not by agonist of EP1, EP3 or EP4. So these findings indicated mPGES-1-derived PGE_2_ exerts a beneficial effect against lung fibrosis through EP2 receptor-mediated signaling pathway, which resembled previous research [31].

TGF-β1 induced myofibroblast differentiation via adhesion-dependent pathways besides Smad pathways. FAK, one of adhesion complexes, is a cytoplasmic protein kinase, and its phosphorylation is related with myofibroblasts differentiation [22,32,33]. In our investigations, we found that butaprost could weaken FAK autophosphorylation, but had no effect on Smad2/3 phosphorylation after TGF-β1 and mPGES-1 siRNA cotreatment in MRC-5 cells, what’s more, FAK inhibitor attenuated α-SMA expression co-administrated by TGF-β1 and mPGES-1 siRNA. These findings indicated that mPGES-1-PGE_2_-EP2 axis’s beneficial effect against TGF-β1-induced α-SMA expression via FAK-correlated adhesion pathway. In addition, the specific mechanism between mPGES-1 and pulmonary fibrosis in patients need to be explored, which will be the key note of our work in future.

## 3. Experimental Section

### 3.1. mPGES-1 Knockout Mice

The mPGES-1 heterozygous mice (mPGES-1^+/−^, C57BL/6 background) were kindly provided by Professor Guan’s Laboratory, with experimental homozygous (mPGES-1^−/−^) and wild-type (mPGES-1^+/+^) mice obtained via inbreeding. Animal experiment protocol conformed to the Institutional Animal Care Guidelines and was approved by the Animal Ethics Committee of Peking University First Hospital.

### 3.2. Animal Groups and Management

Mice were anesthetized with 5 mg/kg pentobarbital sodium (Yunpeng, China) injection, and their tracheas were exposed and instilled with bleomycin (5 mg/kg, Invitrogen, Carlsbad, CA, USA) or saline. After 28 days, the mice were sacrificed and perfused with phosphate buffer saline (PBS) via left ventricle to remove blood from lungs. The left lung was removed for histological examination to evaluate the fibrosis score, and the right lung was used for measurements of hydroxyproline and PGE_2_, for western blotting and real-time PCR (RT-PCR) to quantify related proteins and mRNAs. Twenty-six mice (male, 6–8 weeks old) were randomly assigned into four groups: group 1, mPGES-1^+/+^ mice instilled with saline (*n* = 5); group 2, mPGES-1^+/+^ mice instilled with bleomycin (*n* = 8); group 3, mPGES-1^−/−^ mice instilled with saline (*n* = 5); group 4, mPGES-1^−/−^ mice instilled with bleomycin (*n* = 8).

### 3.3. Cell Culture

Human fetal lung fibroblast cells (MRC-5) were obtained from Basic Research Institute of Peking Union Medical College (Beijing, China), and cultured as previously described [21]. In brief, cells were grown in minimal essential medium supplemented with 10% fetal bovine serum (FBS), essential amino acids, nonessential amino acids and antibiotics, and were maintained at 37 °C in a humidified 5% CO_2_ atmosphere, and medium was changed every 3 days. Cells (3 × 10^5^ cells/well) were then seeded into a 6-well cell culture plate. When cells reached 80% confluence, they were incubated in FBS-free medium for 24 h to synchronize their growth, and then were grown in FBS-containing medium and treated with TGF-β1 (Santa Cruz Biotechnology, Inc., Santa Cruz, CA, USA), PGE_2_, sulprostone, butaprost, CAY10580 (Cayman, Ann Arbor, MI, USA), or FAK inhibitor 14 (Santa Cruz Biotechnology, Inc.). PGE_2_ was dissolved in ethanol, TGF-β1 in citrate buffer, and sulprostone, butaprost, CAY10580 and FAK inhibitor 14 in dimethyl sulfoxide (DMSO).

### 3.4. Knockdown of mPGES-1 Expression in Human Lung Fibroblasts by RNA Interference

We used synthetic siRNA (GenePharma, Shanghai, China) for the experiment. The siRNA sequences for knockdown of mPGES-1 were sense: 5'-GGAACGACAUGGAGACCAUTT, and antisense: 5'-AUGGUCUCCAUGUCGUUCCTT; for negative control were sense: 5'-UUCUCCGAA CGUGUCACGUTT, and antisense: 5'-ACGUGACACGUUCGGAGAATT. MRC-5 cells growing to 80% confluence were synchronized by incubation in FBS-free medium for 24 h and then transfected with 0.1 nmol siRNA-mPGES-1 or siRNA-control using lipofectamine 2000 (Invitrogen). After the transfection for 6 h, TGF-β1 or other reagents were added into the medium for defined hours to study the role of mPGES-1 in the pathogenesis of IPF.

### 3.5. Lung Histological Examination

Lung samples were fixed in 4% paraformaldehyde, embedded in paraffin and made sections of 4 μm thickness. Sections were stained with hematoxylin and eosin (H&E), or Masson trichrome, and assessed pulmonary fibrosis degree by Aschroft scoring method [34].

### 3.6. RNA Extraction and Real-Time PCR (RT-PCR)

Lung total RNA was extracted and reverse-transcribed into cDNA by using a RNA extraction kit (BioTeke, Beijing, China) and following the manufacturer’s protocol (Transgen, Beijing, China). Then 1 μL of cDNA was subjected to PCR in a 25 μL final reaction volume for analyzing the expression of α-smooth muscle actin (α-SMA), fibronectin (FN) mPGES-1, EP1, EP2, EP3 and EP4. The RT-PCR cycling program consisted of a preliminary denaturation (95 °C for 7 min), followed by 35 cycles (95 °C for 30 s, 59 °C for 90 s, and 72 °C for 30 s) and a final elongation step (72 °C for 7 min). Relative gene expression was calculated using the comparative *C*_t_ method [35] to assess the difference in gene expression between the gene of interest and an internal standard gene. Primer sequences and products are listed in Table 1 and the β-actin was used as an internal standard control.

### 3.7. Western Blotting

Fifty micrograms of tissue or cell lysate were resolved in SDS-PAGE and transferred onto a nitrocellulose membrane (Amersham Pharmacia, Buckinghamshire, UK). After blocking in skimmed milk (Applygen, Beijing, China), the membrane was incubated in primary antibody: anti-α-smooth muscle actin (α-SMA, monoclonal antibody, Sigma-Aldrich, St. Louis, MO, USA, 1:1000), anti-fibronectin (FN, monoclonal antibody, Santa Cruz, 1:1,000), anti-β-actin (mouse antibody, Santa Cruz, 1:2,000), anti-mPGES-1 (rabbit monoclonal antibody, Cayman, 1:200), anti-mPGES-2 (rabbit monoclonal antibody, Cayman, 1:500), anti-cPGES (rabbit monoclonal antibody, Cayman, 1:500), anti-EP1 (rabbit monoclonal antibody, Cayman, 1:500), anti-EP2 (rabbit monoclonal antibody, Cayman, 1:500), anti-EP3 (rabbit monoclonal antibody, Cayman, 1:500) anti-EP4 (rabbit monoclonal antibody, Cayman, 1:500), anti-TGF-β1 (Santa Cruz, 1:1,000), anti-p-FAK (CST, Beverly, MA, USA, 1:1,000), anti-FAK (CST, 1:1,000), anti-p-Smad2 (CST, 1:1,000), anti-p-Smad3 (CST, 1:1,000) or anti-Smad2/3 (CST, 1:1,000). Blotted antibodies were developed by appropriate secondary antibodies conjugated with horseradish peroxidase and chemiluminescence. Positive bands were quantified by a densitometer and the β-actin was used as an internal standard control.

**Table 1:** RT-PCR primers and products.

Genes	Sense/Antisense	Primer Sequence (5' to 3')	Products(bp)
α-SMA	Sense	GAAGGAATAGCCACGCTCAG	185
	Antisense	TGCTGTCCCTCTATGCCTCT	
FN	Sense	AATGGAAAAGGGGAATGGAC	134
	Antisense	CTCGGTTCTCCTTCTTTGCTC	
mPGES-1	Sense	CGCGGTGGCTGTCATCA	205
	Antisense	AGGGTTGGGTCCCAGGAAT	
mPGES-1 (H)	Sense	GAAGAAGGCCTTTGCCAAC	200
	Antisense	GGAAGACCAGGAAGTGCATC	
EP1	Sense	TAACGATGGTCACGCGATGG	291
	Antisense	ATGCAGTAGTGGGCTTAGGG	
EP2	Sense	ATGCTCCTGCTGCTTATCGT	126
	Antisense	AGGGCCTCTTAGGCTACTGC	
EP3	Sense	TTGGGCTTGCTGGCTCTG	109
	Antisense	CGTCTCCGTGGTGATTCTGC	
EP4	Sense	GTTCCGAGACAGCAAAAGC	203
	Antisense	CACCCCGAAGATGAACATCAC	
β-actin	Sense	GAGCCCTTTTAGAGCCTT	541
	Antisense	GAGCCCTTTTAGAGCCTT	

H: human. If not indicated, all the primer sequences are referred to mouse origin.

### 3.8. Measurement of Lung Hydroxyproline Content

The hydroxyproline content of the lung for each group was quantified utilizing a Hydroxyproline Testing Kit (Jiancheng, Nanjing, China) according to the manufacturer’s instructions.

### 3.9. Quantification of PGE_2_ in Lung

Thirty micrograms of lung tissue were resolved in 1 mL homogenization buffer (0.1 M phosphate, pH 7.4, containing 1 mM EDTA and 10 μM indomethacin). The samples were homogenized with a sonicator and centrifuged at 5000 rpm speed, and then the supernatant was collected. The content of PGE_2_ and LTC_4_ in lungs was determined using an enzyme immunoassay (EIA) kit (Cayman) according to the manufacturer’s protocols. The PGE_2_ and LTC_4_ content was corrected by the protein content for each sample.

### 3.10. Statistical Analysis

The results were presented as means ± standard error of the mean (SEM). Comparisons were performed using a one-way ANOVA followed by Student’s *t* test, with a *p* value <0.05 considered statistically significant.

## 4. Conclusions

In summary, we firstly reported mPGES-1 deficiency aggravates lung fibrosis after bleomycin exposure in mice. mPGES-1-derived PGE_2_ exerts beneficial effects against pulmonary fibrosis mainly via activation of EP2 receptor signaling transduciton. Activation of mPGES-1-PGE_2_-EP2 signaling pathway may represent a novel strategy for treatment of IPF patients.
